# The contribution of female community health volunteers (FCHVs) to maternity care in Nepal: a qualitative study

**DOI:** 10.1186/s12913-017-2567-7

**Published:** 2017-09-04

**Authors:** Sarita Panday, Paul Bissell, Edwin van Teijlingen, Padam Simkhada

**Affiliations:** 1 School of Health and Related Research (ScHARR), University of Sheffield, Sheffield, S1 4DA UK; 2 School of Health & Social care, Bournemouth University, Bournemouth, BH1 3LH UK; 3 Centre for Public Health, Liverpool John Moores University, Liverpool, L3 2ET UK

**Keywords:** Maternal health, Female community health volunteers (FCHVs), Community health workers, Nepal, Resource-poor countries, Primary health care, Rural, Hill, Terai

## Abstract

**Background:**

In resource-poor settings, the provision of basic maternity care within health centres is often a challenge. Despite the difficulties, Nepal reduced its maternal mortality ratio by 80% from 850 to an estimated 170 per 100,000 live births between 1991 and 2011 to achieve Millennium Development Goal Five. One group that has been credited for this is community health workers, known as Female Community Health Volunteers (FCHVs), who form an integral part of the government healthcare system. This qualitative study explores the role of FCHVs in maternal healthcare provision in two regions: the Hill and Terai.

**Methods:**

Between May 2014 and September 2014, 20 FCHVs, 11 health workers and 26 service users were purposefully selected and interviewed using semi-structured topic guides. In addition, four focus group discussions were held with 19 FCHVs. Data were analysed using thematic analysis.

**Results:**

All study participants acknowledged the contribution of FCHVs in maternity care. All FCHVs reported that they shared key health messages through regularly held mothers’ group meetings and referred women for health checks. The main difference between the two study regions was the support available to FCHVs from the local health centres. With regular training and access to medical supplies, FCHVs in the hill villages reported activities such as assisting with childbirth, distributing medicines and administering pregnancy tests. They also reported use of innovative approaches to educate mothers. Such activities were not reported in Terai. In both regions, a lack of monetary incentives was reported as a major challenge for already overburdened volunteers followed by a lack of education for FCHVs.

**Conclusions:**

Our findings suggest that the role of FCHVs varies according to the context in which they work. FCHVs, supported by government health centres with emphasis on the use of local approaches, have the potential to deliver basic maternity care and promote health-seeking behaviour so that serious delays in receiving healthcare can be minimised. However, FCHVs need to be reimbursed and provided with educational training to ensure that they can work effectively. The study underlines the relevance of community health workers in resource-poor settings.

**Electronic supplementary material:**

The online version of this article (10.1186/s12913-017-2567-7) contains supplementary material, which is available to authorized users.

## Background

Use of Community Health Workers (CHWs) in healthcare provision is increasing worldwide [1–3]. In particular, CHWs have become a central feature of many Primary Health Care (PHC) programmes in resource-poor areas of low-income countries. They are viewed as important contributors to achieving the Millennium Development Goals (MDGs) for maternal and child health [1, 2, 4]. National and international decision-makers are turning to CHWs to strengthen PHC and to support post MDGs – Sustainable Development Goals – which aim to provide universal access to reproductive healthcare [1, 3–5]. While some CHW programmes are small independent projects, others are large nationwide programmes managed by government agencies, such as Female Community Health Volunteers (FCHVs) in Nepal. FCHVs are the lowest level or ‘first contact’ PHC providers within the public healthcare system.

Over the last two and half decades, Nepal has experienced a significant reduction in maternal mortality despite continued poverty, political conflict and a limited provision of universal healthcare services [6–8]. Despite high home delivery rates (72%) and a low rate of skilled attendants at birth (36%) [9], Nepal reduced its Maternal Mortality Ratio (MMR) by 80% from 850 to an estimated 170 deaths per 100,000 live births between 1991 and 2011 and achieved the MDG Five [10], compared with a 47% decline worldwide [11]. The remarkable reduction in MMR has also been accompanied by improvements in women’s education coupled with the increased use of contraception and the associated decline in fertility rates [12]. Some 52, 000 FCHVs working across the country distribute temporary contraception or offer referrals for other methods of family planning [13]. These volunteers are often credited for the improvement in maternal health in rural Nepal [6, 10, 14] but there is no empirical evidence to support this.

FCHVs are typically local women above 25 years of age who receive a basic 18 days of training in various PHC topics, including maternal and child healthcare services [15]. Although FCHVs are expected to work part-time with an average of five to six hours work per week [13], it may vary depending on the programmes in which FCHVs are involved. FCHVs appear to be committed to volunteering as their retention rate is very high (96%) [13]. Social respect, religion and moral duty were reported as key reasons for such high motivation, but this was mainly the policy-makers point of view [16]. The voices of these volunteers in policy-making are missing. The fact that all the volunteers are female demands further research because female CHWs are more likely to work unpaid than their male counterparts indicating a huge level of gender disparity [17, 18].

Before the beginning of the FCHV programme in 1988, basic maternal and child health care including family planning services was provided by a group known as community health leaders. They included both male and female volunteers but the males were not easily accepted by female service users. Because of this the male volunteers were replaced by female volunteers or FCHVs, who are married and are mothers. Female service users often find it easier to discuss their pregnancies and childbirth with female volunteers as shown by Feldhaus et al. [19]. In addition to the unpaid FCHVs, there are paid CHWs who work full-time and can be male (village health workers) and female (maternal and child health workers), but FCHVs are the key PHC providers in villages.

FCHVs’ service provision is not without challenges. Mothers may not use their services if they feel that the service quality is poor, as shown by a study in remote Nepal which found that almost 67% of women never sought services from FCHVs [20]. Services of CHWs are also affected by health systems factors, such as the availability of training, supervision, access to medical supplies and provision of monetary and non-monetary incentives, which are often challenging in poor-income countries [1, 3, 21]. Despite the challenges, the role of FCHVs have been extended to include a wide range of promotional, preventive or curative healthcare services [22–25].

The main role of FCHVs is to facilitate mothers’ groups meetings, where women from local villages gather and discuss health topics. FCHVs refer pregnant women or mothers for health checks, provide iron tablets and distribute misoprostol to prevent post-partum haemorrhage. They also maintain a record of health activities and report this to local health facilities [26]. The growing use of FCHVs in the provision of maternity care for the rural population means that understanding the FCHVs’ view is important. This is because the development of CHW programmes and policies requires the communication of knowledge that includes the views of people working on the ground [27]. However, little is known about the views and experiences of FCHVs [28, 29] and how the work of FCHVs is viewed and experienced by service users and local health workers. This study aims to provide subjective insights into the role and experience of FCHVs. While some issues found in our study might be specific to particular Nepalese villages, others are common to a wider range of settings. We assess how the inclusion of FCHVs in a government healthcare system facilitated the provision of maternity care in two different contexts and the challenges they faced undertaking their role as volunteers.

## Methods

### Participants and procedure

The majority of the Nepalese population live in Terai, a lowland region along the southern border of the country (50.3%), and the hill regions (43%), with fewer living in the mountain regions (6.7%) [30]. Due to the low population density and likely resource issues in locating potential participants in the mountains, this area was not included. Data were collected between April and September 2014. Villages from the hill and Terai were chosen purposefully for inclusion in this study. In the hill region, villages from Dhading were selected because the district had regular supervision of FCHVs and mothers’ group meetings [26]. The district was connected to the capital, yet the study communities remained isolated with relatively limited access to healthcare services. In addition, SP’s familiarity with the place and the diversity of the villagers were further reasons for the selection of these villages.

In Terai, villages from Sarlahi were selected because this district has relatively easy access to healthcare services while comprising an ethnically diverse population. The main study participants were FCHVs operating at ward-level – the smallest unit of the village. Interviews were also conducted with service users or potential service users (pregnant women or mothers of children under two), and local health workers who were supervising or supporting FCHVs. Understanding their views was core to understanding the volunteers’ work and complemented the views of volunteers.

The study received ethical approval from the Nepal Health Research Council. SP explained the purpose of the research to participants and informed them that their participation was voluntary and the interviews would be recorded using a digital audio recorder. Participants were also told that they would not be identifiable once the interviews were transcribed. In some cases, informed consent was obtained verbally, since some participants were illiterate. In focus groups, the participants were advised at the initiation of the session that what was discussed within the group would not be shared outside of the group.

A semi-structured topic guide was used to ensure that key research questions were addressed in every interview and focus group discussion while also allowing flexibility to bring up any new issues identified by the participants. An additional PDF file shows this in more detail (see Additional file 1). The aim of the focus groups was to capture the breadth of perception and experiences of FCHVs through group interaction rather than to develop consensus [31]. SP conducted both interviews and the focus groups with the FCHVs with varying years of work experience. She tried to ensure that her position as a female from an upper Hindu caste family with a high level of education would not have an impact on the participants’ complete disclosure of events. This is because social class of the interviewer can influence the subject’s answers [32]. Instead, SP’s community health work experiences in remote Nepal and her background as a nurse might have given participants a feeling of being at ease, because women in general prefer to share their issues with female health workers.

Interviews were mostly conducted in participants’ homes but some interviews and group discussions were conducted in health centres where volunteers often gathered for training or reporting. Interviews lasted from 15 to 60 min. Some participants were uncomfortable about being recorded and asked for some comments not to be recorded. In such situations, the use of field notes was particularly relevant to remind SP of important points that could be useful for interpretation or analysis [33] and which would also enrich the primary data.

### Analysis

SP developed a set of thematic codes for the interview and focus group discussion data by reading and re-reading transcripts. All the transcribed data were then transferred to NVivo10, and coded using these initial themes. Additional codes were also identified as the analysis proceeded. EvT coded four interviews and a group discussion transcript and compared this to that undertaken by SP to ensure data quality [34]. Codes were compared and discrepancies were discussed prior to further analysis. Thematic analysis was used because of its ability to identify and recognise the visible and the underlying themes in the data [35, 36].

Data were analysed in an iterative fashion, moving back and forth between transcripts, reflective notes, field notes, and the literature. The reliability of coding and interpretation was also checked during analysis by re-examining the transcripts. Common themes across the data set were developed by merging data from different data sources, a technique known as data triangulation [37]. Data were triangulated in three different ways: a) groups of interviewees (FCHVs, their services users, and the local health workers), b) research methods (interviews and focus groups) and c) study sites (the hill and Terai regions). Data triangulation made the data analysis more comprehensive allowing a broader understanding of FCHVs’ work in maternal healthcare.

## Results

The main research findings are organised under four main subheadings: a) the profile of the research participants; b) immediate access to public healthcare resource in remote villages; c) public health centre support for the use of locally appropriate approaches; and d) perceived self-empowerment of volunteers despite monetary issues.

- Profile of research participants
  Table 1 summarises the data collection methods and the types and numbers of study participants by location. Of the 75 participants in this study, semi-structured interviews were conducted with 20 FCHVs, 26 users or potential service users (females) and 11 local health workers (6 males and 5 females) who supervised FCHVs’ roles. Four focus groups were held with 19 FCHVs. A FCHV, who had been interviewed, also participated in a focus group, thus making the total number 75 (Table 1). The majority of FCHVs (*n* = 11) interviewed were aged 45–59 years compared to the national average age of 38 years ([13]). Most FCHVs (27/39) had been working for more than 10 years, with a range from 1 to 26 years.
  Eight volunteers were illiterate, while seven others had received adult education meaning that they could read and write Nepali. The remaining FCHVs had some school education.
  Most of the service users (19/26) were married by the age of 20, with eight married by the age of 15. In Terai, all women except one had delivered their children in health centres, while in the hill villages six of them had had home deliveries.
  Public healthcare services were relatively accessible in Terai, taking approximately 30 min to reach on foot, compared with the hill region where the walking distance to the nearest health centre ranged from 40 min to an hour and in one case up to six hours. Out of the total 11 health workers interviewed, seven worked in public health centres and the remainder were from non-governmental organisations.

**Table 1:** Participants in interviews and focus group discussions

Study methods	Types of study participants	Number of participants by location			Total
		Hill (Dhading)	Terai (Sarlahi)	Kathmandu	
Interviews	FCHVs	8^a^	12	-	20
	Pregnant women/mothers	14	12	-	26
	health workers (public)	4	3	-	7
	health workers (private)	2	2	-	4
Focus Groups	FCHVs	4^a^	11	4	19
Total		32	40	4	75^a^

^a^One FCHV (Female Community Health Volunteer) in the hill village was present in both interview and focus group discussion which made the total number of participants 75

**Fig. 1:**
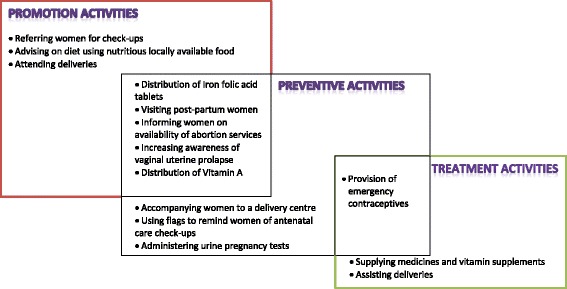
Summaries of Female Community Health Volunteers’ (FCHVs) maternal health services

b) Immediate access to public healthcare resources in remote villages
  Data analysis showed that all health workers, a majority of service users and all FCHVs acknowledged the volunteers’ role in the provision of basic maternity care in villages. The fact that a FCHV was accessible every day of the week at all hours and usually lived within walking distance meant that service users could easily approach her when needed. The FCHVs’ main role was to identify and refer pregnant women to attend healthcare services in order to avoid potential complications during home births in the absence of a skilled birth attendant. They also provided iron tablets to pregnant women and asked them to have local food full of nutrients. When required, FCHVs accompanied pregnant women while visiting health centres or during home deliveries. In so doing, the links with and support from government health centres remained important.
  Service users’ views on FCHVs were mixed. In Terai, the FCHVs’ role was more limited, as they received less support from healthcare centres and there was also relatively easy access to formal healthcare services, and thus the perception of them by service users was rather less positive. However, in the remote hill villages, service users usually appreciated the FCHVs services as they regularly shared health information on the importance of regular health checks during pregnancy and health centre deliveries. A service user commented:
*“We have a mothers’ group in our village. In that group, we are often given training. There is a FCHV who shares information on the importance of going to the health centre for childbirth. Now all the sisters do not stay at home, but go to health centres for check-ups and to give birth in hospital.” WomanD11.*
  The FCHVs were often the only trained government healthcare workers immediately accessible in the hill village. A FCHV commented:
*“If women call at night, I go there immediately. In the last 2-3 deliveries I attended, I did not get home from the regional hospital until 1am. Last time, I was suddenly called to attend a woman ...While there, the woman began to bleed and shortly thereafter she went into labour. I was half-dressed right throughout the night.” FCHVD8.*
  The FCHVs also informed service users of available free healthcare services and transportation allowances for delivering in the government healthcare centres. However, the cash payment for transportation allowances was often delayed and was less than the actual cost incurred. This was because a substantial amount of money for transportation costs was required if pregnant women were to be carried to the health centre from the remote hill villages. As a result, many women in the region continued to deliver at home. A health worker explained:
*“During the delivery, NRs 1,000* (about £6.66) *is available in the hill communities, but if someone visits from ward number 9, then it costs at least NRs 8,000* (£53.33) *to NRs 9,000* (£60) to get to the health centre. *The patient needs to be carried up and down steep hills for anywhere from 10 to 12 hours. During this time, a patient may be required to buy food which may bring the total to as much as NRs10 to 12,000* (£80). *Not all people can afford this.” HW4.*
  It was also reported that the inability of villagers to afford the associated cost of childbirth combined with the lack of skilled healthcare providers in a remote village of Dhading meant that the FCHVs often had to attend to women during childbirth. In one case, a pregnant woman with nine children could not manage the six-hour walk to the nearest health centre; as a consequence, the FCHV had to work beyond what is expected of her. Once the woman went into labour, she assisted with the delivery despite the mother encountering complications involving a uterine prolapse:
*“It is better to become insane rather than die. We needed to treat in whatever way we could. I went to her and I tried to make her cool with a hand fan, and gave her some liquid food. I found that the placenta had been retained inside. Then I moved the placenta slowly and took it out. Her cervix was completely damaged. I told her, ‘You had 10 babies, that’s why your uterus protruded out.’ I asked her to sleep and not to stand for some days and to eat lying down without feeling shy. I saved her life.” FCHVD4.*
  A majority of the FCHVs usually served the poor section of the populations and admitted that the local health services are for the poor, as richer people directly accessed services from higher level health centres:
*“Rich people go to Kathmandu. Usually poor people go to xxx (PHC) and the ones who are ultra-poor, they come to us.” FCHVD8.*
  All interviewed health workers credited the FCHVs for their contribution to maternal health improvement and praised their efforts to increase the number of women attending health centres. A health worker from a hill village commented:
*“When we started the birthing centre, there were only two cases of delivery in the first year. After the implementation of a health awareness campaign through FCHVs and the mothers’ groups, the number of health centre deliveries rose to 60 cases within a year.” HW5.*
  A majority of health workers admitted their reliance on the FCHVs reporting health activities at the community level:
*“FCHVs are the ones taking responsibility for everything at the community level. The health record brought by FCHVs is forwarded to the upper health centres. In other words, we are sharing our work with them and our earning is partly possible because of them.” HW8.*
  However, many health workers and FCHVs reported that the illiteracy of the volunteers presented a challenge as it caused difficulties in reporting health activities. A FCHV commented:
*“There is a girl [name]. She helps to record health information. Otherwise, I ask sisters [female health workers] to write the report for the health centre. I verbally report to them.” FCHVD4.*
  Fig. 1 summarises the FCHVs’ maternal health activities. The FCHVs were involved in maternal health promotion, disease prevention and treatment activities.
c) Public health centre support on the use of locally appropriate approaches
  Data analysis showed the major difference between the FCHVs in the two regions studied was the support available from government health centres. Whilst the FCHVs in Terai reported minimal support, the FCHVs in the hill villages were trained to address locally relevant issues, had access to medical supplies and were encouraged to use locally appropriate strategies to deliver key maternal health messages. Consequently, the FCHVs in the hill villages reported additional healthcare activities such as distributing medicines, administering pregnancy tests and informing women of the availability of emergency contraception or legal abortion services. They shared health information informally, for instance by singing folk songs with health messages in them or visiting new mothers with food hampers. Some examples are illustrated below.
  In the hill villages, FCHVs treated people with simple illnesses such as headaches, fevers and stomach aches. They could easily do so, as public health centres provided them with some basic medicines (antacids, metronidazole and paracetamol tablets, vitamins). One FCHV commented:
*“I have metro [metronidazole]. I give that to treat stomach ache as it cleans our stomach. I bring the medicine from the health centre and give it to the people who need it. Metro is given for diarrhoea, cetamol [paracetamol] is for fever and vitamins [vitamin B complex] are for weak people.” FCHVD1.*
  However, in the majority of cases, the FCHVs working in Terai could not provide any medicine. This included iron tablets for pregnant women or mothers since the health centres lacked a sufficient supply. As a result, visits to pregnant women were curtailed, as illustrated below:
*“There are no iron tablets. Health workers ask me to bring a record of pregnant women. If I don’t have iron tablets with me, what reason can I give to visit them? At least, after recording her pregnancy details, I could have given her some iron tablets and asked her to visit a health centre. We have 6-7 pregnant women in the village and I have not been able to visit them.” FCHVS20.*
  It is rare for a FCHV to give injections, apart from specially trained cases, but a FCHV from Terai reported an activity that she was not authorised to do. She reported that she was giving Tetanus Toxoid (TT) injections:
*“I have a small shop at my home. I sell medicine. Some children even receive injections. If someone comes with a cut, I give some medicine and bandage…I give TT. I give all the injections. One thing, I don’t do is inject on the nerves [She indicates the place on her upper arm as an injection site].” FCHVS13.*
  A majority of the FCHVs in the hill villages reported using informal approaches to share key maternal health messages including information about danger signs and symptoms such as bleeding, fever and swelling of limbs. They contributed to festivals or gatherings to inform women about the importance of health checks:
*“The volunteers sang in a folk rhythm that conveyed health messages on antenatal care visits, and recognition of danger signs during pregnancy - bleeding, body swelling, and fever.” (FGD2, 31st May 2014).*
  Some FCHVs reported that they visited new mothers, who came from poor economic background, with a food hamper prepared from donations from mothers’ group members. The visit also provided an opportunity to check the health status of mothers and babies. One service user commented:
*“In our mother’s group, there is also a programme of visiting a new mother. We visit her with around half kilogramme (kg) of ghee or oil, and 10 kg of rice.” WomanD11.*
  Another important role of the FCHVs was to inform women of the availability of abortion services. Educated volunteers in the hill villages reported that they administered urine pregnancy tests and counselled women on using emergency contraception or visiting antenatal care check-ups. A FCHV reported providing emergency contraception too. Such services were important for women with unexpected pregnancies:
*“For women who have many children and whose menstruation has stopped, we perform urine check-ups. Then, we ask them about the length of time since they had their last menstruation. If they have had enough children already we tell them to go* [for abortion] *because till the ninth week of pregnancy, the baby can be aborted by using medicine.” FCHVD5.*
d) Perceived self-empowerment of volunteers despite monetary issues
  While all the FCHVs expressed dissatisfaction with the monetary incentives they received, they expressed their commitment to volunteering. One of the key reasons for the FCHVs to volunteer was their reported experience of self-empowerment through volunteering. In particular, FCHVs reported that they appreciated the opportunities to learn new knowledge and skills. They also positively valued travelling outside their houses, meeting new people, and especially gaining respect from health workers for the work they undertook. Some examples are illustrated below.
  The majority of volunteers welcomed the respect and recognition they gained as a volunteer:
*“All people in the village recognise me as a FCHV. I feel happy being able to serve children under the age of five and pregnant women.” FGD3 participant5.*
  One FCHV commented on the importance of sharing knowledge and its effect on her:
*“During training, we get the opportunity to meet in one place and share each other’s experiences…Being a FCHV provides a reason to leave my home and I feel good while attending the training. This motivates us at our work.” FGD3 Participant 1.*
  All health workers in the study praised the role of the FCHVs in maternal health. Such praise encouraged the FCHVs to volunteer. A health worker commented:
*“FCHVs have been providing health information to pregnant women and mothers in their communities. This has produced a big change. On the whole, FCHVs play a great role in the reduction of maternal and neonatal deaths.” HW5.*
  However, many FCHVs highlighted a monetary problem while attending training or other health activities. One FCHV mentioned that she needed to pay someone to do her work while she was volunteering:
*“We need to have someone to replace us in doing farm work during our training days. It is too difficult [addresses interviewer]. We get NRs 200 (£1.33) per month, but hiring someone to work at our farm is not possible at that price. We cannot even get the workers at NRs 500 (£3.33) per day. It’s too difficult.” FGD2participant1.*
  Health workers also agreed that the volunteers need to be compensated, as they are given additional responsibilities without any reimbursement. A health worker mentioned that often the FCHVs had to walk a long distance to attend meetings without any reimbursement for their time particularly in remote villages:
*“From Sertung village, it takes 4 hours to reach the other village. The government provides only NRs 200 (£1.33) per day where the volunteers have to walk almost 8 hours a day to attend the training.” HW4.*
  Despite being a volunteer, some volunteers in the both regions expressed that they wanted salaried work:
*“After all the work we do we should be paid. We create all the records. Even educated people don’t work much. For example, health workers just use the report that we have given to them.” FCHVD6.*
  Although volunteers complained that they lacked monetary support to undertake their activities and health workers also agreed with this view, all the volunteers were committed to continuing volunteering. This was mainly because the volunteers perceived that they were benefitting from the work as a volunteer and saw themselves as empowered women.

## Discussion

This study has provided insights into the experiences of the FCHVs in volunteering in the provision of maternity care. While there have been previous studies of experiences and perceptions of CHWs, our study is the first of its kind from Nepal; bringing together perspectives from the FCHVs, their service users and paid local health workers; and focusing on two geographically distinct regions – the hill and Terai. While Dhading is a hill district with better resources and better connections to referral networks and Kathmandu (the capital city) than most of the hill districts in the Central and Far Western regions, some study villages remain isolated with relatively difficult access to healthcare services. The main finding of the study is that the FCHVs supported by the government healthcare system can deliver basic maternity care in resource poor areas as seen by their work in these two settings. The FCHVs are by necessity providing services beyond expectations – assisting childbirth, distributing medicines and emergency contraception – that are elsewhere undertaken by professional health workers. As provision of facility-based childbirth services in resource-poor areas is still beyond the reach of many resource-poor countries [38], the findings presented here illustrate how the FCHVs facilitate maternal healthcare provision in such settings, including the challenges they face and potential solutions that might be externally applicable.

The FCHVs reported that they assisted with deliveries although technically they were not supposed to do so. In doing so, they bridged the gap between the poor health care provision and the community, as Nepal lacks sufficient numbers of professional midwives to deliver such services [39]. This situation is unlikely to change in the near future due to difficulties recruiting and retaining midwives or doctors in the villages, since they often choose to live in urban regions and work in the private sector ([40], [41]). Therefore, the FCHVs involvement in childbirth was essentially a necessity rather than an option in the geographically inaccessible areas [42]. Similar findings have been noted in Afghanistan and Pakistan where the poor accessed healthcare services from the CHWs, while wealthier groups were able to use skilled attendants [43, 44]. This indicates that the services of CHWs can be beneficial in resource-poor areas because they allow people to receive at least basic healthcare services.

We found that the main difference between the two study regions was the support available to the FCHVs from government health centres. Compared to the FCHVs in Terai, the FCHVs in the hill villages were relatively well supported in terms of training, supervision and access to medical supplies such as paracetamol, antacids, metronidazole and vitamin tablets so that they could treat some minor ailments such as headaches, diarrhoea and fevers. Earlier studies suggest that if CHWs were able to provide medicines to the villagers, even if it was only vitamin tablets, then the village population were likely to place their trust in them [45, 46]. However, if the volunteers are not trained then their actions can be harmful as in the example of a volunteer giving injections in our study. While our study shows the possibility of expanding the role of volunteers in medicine distribution, contextual and health system factors need to be considered for the programme to function as seen in Ghana [47].

Unlike FCHVs in the hill region, the FCHVs in Terai could not provide any medicines as the health centres lacked a sufficient supply. This not only reduced volunteers’ regular health activities, but also reduced their morale as seen by some volunteers’ unwillingness to visit pregnant women. In addition, the illiteracy of some volunteers meant that they reported information verbally. Health workers often did not verify the information and saw the volunteers as information providers. A lack of connection between healthcare centres and the FCHVs was seen which if it continues, threatens the sustainability of the programme. For the CHWs within government system to work, the government not only needs to own the programme and makes strategies for its implementation [47], but it also needs to ensure that the implementation of the programme is effective. The support of the health system is thus extremely important to the morale and therefore the activity and interest of the volunteers.

Our study also showed that the FCHVs used innovative approaches to the delivery of health information, such as sharing health messages through local songs and visiting new mothers with food hampers. Such local practices could be useful ways to raise awareness about danger signs during pregnancy, childbirth or post-partum; and to inform women of free healthcare services at public health centres. In doing so, the expectation is that pregnant women could attend health centres with the capacity to manage complicated deliveries, which is another challenge in resource-poor areas [38]. Nonetheless, the importance of informal sharing of maternal health messages by the FCHVs is highly relevant in rural regions and has been reported in other maternal health programmes [48].

We found that money was one of the key concerns of FCHVs, who clearly expressed the need for monetary compensation for volunteering. The volunteers’ domestic responsibilities often mean that they do not have the same amount of time for volunteering. While volunteers are increasingly expected to deliver a range of PHC, financial support for these groups remains limited. Yet, FCHVs sometimes had to pay out of their pocket and often did not receive reimbursement for their expenses. In addition, as the volunteers are mostly poor and overburdened, asking them to provide their services for free prevents them from working and is not sustainable [25, 49]. If CHWs are expected to provide regular and effective maternal healthcare services they should therefore be remunerated as shown by a growing volume of research in South Asia and Africa [1, 50–54].

Policy-makers in Nepal perceived that paying volunteers is not feasible [16] which could be due to the assumptions that women can volunteer their time as seen in the lay health worker policy development in South Africa [55]. The belief that women can volunteer freely can also be related to the lower decision-making power of women than those of men [18, 56, 57]. The dependency on female volunteers to support weak healthcare systems not only affects the volunteers at an individual level but also reinforces gender inequalities in the whole society [17]. Further research is needed to include details of gender aspects within the health research so that underlying power relations and its effects can be explored [18].

We also found that despite the challenges, all the FCHVs were committed to volunteering. Indeed, one of the key reasons for their continued willingness to volunteer in these roles was their perceived self-empowerment. The empowerment they described was related to opportunities for training and education as a result of volunteering, which enabled them to take care of themselves as well as the villages. This is consistent with other studies ([58], [59]) that showed that volunteering provided volunteers with skills which other members of their community group did not have. Such skills are especially important for women in rural Nepal, as they are rarely involved in household decision-making [56, 57]. Similar findings were reported in Bangladesh and Ethiopia where CHWs volunteered because of their desire for self-development [54, 60].

This study was conducted in specific areas of Nepal and therefore there are potential dangers in extrapolating findings across the whole diverse country. While this research sheds light on the subjective experience of FCHVs, no research to date has been able to demonstrate that the FCHVs roles themselves have an impact on maternal mortality or other health outcomes; quantitative studies are needed to do this. Yet qualitative methods is the best method for exploring people’s experiences or perspectives [61]. The multiple methods of data collection, interviews, FGDs and field notes; and the use of triangulation enabled the researcher to include more comprehensive views of study participants. While most interviews went as planned, a few were interrupted. A FCHV who had been interviewed also participated in a focus group (Table 1) despite SP’s attempt to stop her involvement. The FCHV was enthusiastic to contribute to the group discussion. Sometimes participants’ family members or neighbours often interrupted the interviews as they were generally held on verandas outside houses. Questions directed at mothers or pregnant women were answered by either their husbands or mothers-in-law, who often influenced the uptake of pregnancy care services by these women, as reported in the literature [57, 62]. In order to avoid such interruptions during the interviews, SP explained the importance of interviewing women on their own and wherever possible, she went out with the interviewees to the kitchen gardens or the open fields.

The research findings provide detailed insights into the subjective accounts of the FCHVs’ experiences in maternity care in two different settings within Nepal. We recognise that this paper does not cover the issue of trust and how this is negotiated between the FCHVs and the women they serve and the health system they represent, but that this will be explored in more detail in a subsequent paper. The findings from the current paper can be utilised to provide insights into future policy and programme decisions that the Nepali Ministry of Health and Population and other officials can then use to determine how best to move forward with the FCHV programme, as central level policy-makers are grappling with these challenges [25]. In addition to the local relevance of this research, it is likely that aspects of the discussion presented above have relevance and are transferable elsewhere in Nepal as well as in similar resource-poor settings elsewhere.

## Conclusions

The evidence from this study demonstrates that FCHVs, supported by the government healthcare system, play an important role in the provision of basic maternal healthcare in resource-poor settings in Nepal. As seen by the different working capacities of the FCHVs in the two study regions, their role continues to be highly relevant in remote villages, which have poor maternal health outcomes ([7, 9]). In the absence of immediate access to healthcare, volunteers shared basic maternal and child health information and referred women for health checks or delivery. While the FCHVs in Terai could not function well and reported reduced work interest, the FCHVs in the hill region served beyond what was expected of them. For example, they assisted with childbirth, provided emergency contraception and distributed medicines. Their involvement in medicine distribution provides a compelling case for further exploration of expanded roles for community-based workers. In both study regions, monetary compensation for the FCHVs was by far the biggest concern and needs to be considered seriously if we want the FCHVs to remain motivated and continue working to improve maternal health in resource-poor areas. The good aspect of the FCHV programme was the volunteers’ perceived self-empowerment through volunteering, which kept them motivated at work.

This paper offers an important opportunity to hear directly from the FCHVs, who are the foundation of the Nepalese public healthcare system and provide PHC services to its rural communities. The benefits to women of the volunteers’ work were significant, as a result was that more pregnant women and mothers from the poorest communities were aware of existing healthcare services and would visit health centres, thereby filling the gap in service provision. In addition, the health awareness of these women volunteers is a substantial public health benefit. Their insight can facilitate programme and policy efforts to reach women in remote regions and to achieve universal healthcare coverage for maternal health thus possibly leading to further improvement in maternal health. We believe that the study findings have implications for other similar CHWs in resource-poor settings.

## Additional file

Additional file 1: Interview and focus group discussion guides. The file contains information on the questions and probes used in the interviews and focus group discussions. (PDF 168 kb)

## Abbreviations

CHW: Community Health Worker
FCHV: Female Community Health Volunteer
MDG: Millennium Development Goal
PHC: Primary Health Care
