# Supplementary material for: The contribution of female community health volunteers (FCHVs) to maternity care in Nepal: a qualitative study
Source: BMC Health Serv Res. 2017 Sep 4;17:623. doi: 10.1186/s12913-017-2567-7 (PMC5584032; doi:10.1186/s12913-017-2567-7)
Supplement: Additional file 1: — Interview and focus group discussion guides. The file contains information on the questions and probes used in the interviews and focus group discussions. (PDF 168 kb) [file 12913_2017_2567_MOESM1_ESM.pdf]

Topic Guides for Data Collection

1. Topic Guide for semi-structured interviews

General information of Female Community Health Volunteers (FCHVs)

Age

Gender

Caste/Ethnicity

Education

Work experience in years

Religion

Husband's occupation

Number of houses covered

Distance from the nearest government health centre

Topic Guide for semi-structured interviews with FCHV

- I am interested to know about your role as a FCHV. Tell me about your experience of being a FCHV (or describe your typical working day/what kinds of work do you generally carry out?)
- What services do you provide in relation to maternal health? (It seems sharing health messages to groups is important part of your function. What types of health messages do you disseminate? How do you inform mothers?)
- What was the state of maternal heath in your village before you became FCHV? What difference have you found since you started?
- What have you done to make pregnant women to attend health care check-up?

- What is your role during delivery of women? What have you done to make women to deliver at health centre?
- What do you say to the women who have recently delivered?
- How have you been able to reach women or pregnant mothers in the village), if not why not? (Mothers' group meeting/ home visits/referred by someone..)
- What are the difficulties/barriers you faced while delivering maternal healthcare services?
- Do women or community members seek or accept your services? Yes/no, why? (Your selection process, training, skills, communication, access, availability)
- How are you supervised at work? When do the supervisors come to you for supervision?
- What kinds of support system available for you to deliver these services? (Skills, training, supervision, regular access to supplies, incentives, availability of nearby health facilities)
  What types of practical things are you given with?
- Why did you become FCHV? What made you to serve mothers and children? (motivations)
- What makes you motivated to continue this work?
- In your opinion, what is necessary for FCHVs to enable them to deliver standard services to the women in village that helps in maternal health improvement?

At the end of an interview, ask:

Would you like to add anything?

Topic guide for interviews with women (service users or potential service users)

- Where do you go when you do not feel well? Why do you go there?

- Have you received services from a FCHV?
- What kinds of services have you received from FCHVs?
- Why do you seek care from her? / What makes you to seek services from her?
- How do you access maternal health services from FCHVs and why do you use it this way? How FCHVs help you to seek antenatal, delivery or postnatal care services from health centres?
- Does a FCHV come to you to provide services, (or do you visit her)? Why did you see her last time?
- What is your impression on FCHV’s services? (What do you think about the relevance of their services?)
- What makes FCHVs’ opinion important to you? (Looking for their acceptance)

At the end of an interview, ask:

Would you like to add anything?

Topic guide for interviews with local health workers

- What do you think about the role of FCHVs in maternal health improvement in your community?
- Why FCHVs are motivated to volunteer? Why older volunteers are committed to continue volunteering?
- What challenges FCHVs face in carrying out their activities?
- What sorts of training FCHVs have been provided with in relation to maternal health and whether the training to FCHVs is adequate to deliver assigned services? (For example whether FCHVs have been provided with training on community mobilisation, birth preparedness packages, communication skills, counselling skills).
- How do you supervise them (including mothers' groups meetings and monitoring of FCHVs work)?
- What can be done to improve FCHVs' activities?

  Would you like to add anything?

2. Topic guide for focus group discussions with FCHVs

- What kind of work you generally do? (Could you explain more about maternal healthcare services?)
- What helps you to deliver these services?
- How have you been able to reach women in the village, if not why not?
- Why they (women or community member) accept your services? (Your selection process, training, skills, communication, access and availability etc.)
- If the service users are not willing to accept your services, what are the reasons for this?
- What challenges do you face while providing maternal healthcare?

At the end of focus group discussions, ask:

Would you like to add anything?
